# Non-Negative Matrix Factorization for the Analysis of Complex Gene Expression Data: Identification of Clinically Relevant Tumor Subtypes

**DOI:** 10.4137/cin.s606

**Published:** 2008-05-29

**Authors:** Attila Frigyesi, Mattias Höglund

**Affiliations:** 1 Department of Cardiology, Lund University Hospital, SE-221 85 Lund, Sweden; 2 Centre for Mathematical Sciences, Mathematical Statistics, Lund University, SE-223 62 Lund, Sweden; 3 Department of Clinical Genetics, Lund University Hospital, SE-221 85 Lund, Sweden

**Keywords:** NMF, gene expression, tumor classification, metagenes

## Abstract

Non-negative matrix factorization (NMF) is a relatively new approach to analyze gene expression data that models data by additive combinations of non-negative basis vectors (metagenes). The non-negativity constraint makes sense biologically as genes may either be expressed or not, but never show negative expression. We applied NMF to five different microarray data sets. We estimated the appropriate number metagens by comparing the residual error of NMF reconstruction of data to that of NMF reconstruction of permutated data, thus finding when a given solution contained more information than noise. This analysis also revealed that NMF could not factorize one of the data sets in a meaningful way. We used GO categories and pre defined gene sets to evaluate the biological significance of the obtained metagenes. By analyses of metagenes specific for the same GO-categories we could show that individual metagenes activated different aspects of the same biological processes. Several of the obtained metagenes correlated with tumor subtypes and tumors with characteristic chromosomal translocations, indicating that metagenes may correspond to specific disease entities. Hence, NMF extracts biological relevant structures of microarray expression data and may thus contribute to a deeper understanding of tumor behavior.

## Introduction

Several powerful tools for the analysis of gene expression data have been developed. Typically, methods such as hierarchical cluster analysis and principal component analysis focus on the dominating structures in the data and fail to depict alternative features and local behavior. In addition standard clustering techniques based on correlated behavior group genes into non-overlapping clusters, less optimal from a biological point of view as individual genes may take part in different cellular responses. To explore alternative methods to interpret complex gene expression data we recently applied independent component analysis (ICA) (Frigyesi et al. 2006). Even though this method could identify additional substructures in the data compared to standard gene clustering methods, ICA, as well as PCA basis vectors have both positive and negative coefficients. Thus modeling of the original data may involve complex cancellations between positive and negative values. In a gene expression situation negative values contradict physical realities as a gene with a negative expression cannot be readily interpreted; in a biological setting a gene is either present with a variable intensity or it is absent. It therefore makes sense to constrain both the factors and their coefficients to a non-negative setting. One such alternative approach is non-negative matrix factorization (Lee and Seung, 1999) in which data is modeled as the product of two non-negative matrices. Thus NMF reproduces data by only additive combinations of non-negative vectors. Lee and Seung (1999) showed that the basis vectors obtained by NMF when applied to facial images were “non-global” and composed of “natural” parts of the facial images. The non-negativity requirement restricts the basis vectors to be composed of co-activated pixels from the same part of the face. This is attractive from a microarray analysis perspective as such co-activation may be equivalent to expression modules or metagenes. Furthermore, as NMF detects local behavior of variables (genes) this method may detect gene sets that show correlated expression in subsets of tumors but not in others, patterns that would be undetected by approaches based on global gene behavior. Kim and Tidor (2003) applied NMF to 300 yeast whole genome expression analyses and could show that the obtained basis vectors were stable and that vectors could be annotated by MIPS functional categories. Several modifications of the original NMF algorithm have been presented (Brunet et al. 2004; Gao and Church, 2005; Carmona-Saez et al. 2006; Wang et al. 2006; Fogel et al. 2007, Kim and Park, 2007). In the present investigation we evaluate NMF as a tool to retrieve significant biological information from complex microarray data and focus on the biological interpretation of the metagenes thus obtained. We show that several obtained metagenes correlates with tumor subtypes and tumors with characteristic chromosomal translocations, indicating that metagenes may correspond to specific disease entities. Hence, NMF extracts biological relevant structures of microarray expression data and may thus contribute to a deeper understanding of tumor behavior.

## Materials and Methods

### Data sets

The CNS data (Pomeroy et al. 2002) was downloaded from supporting information to Pomeroy et al. (2002) to contain 34 cases; 4 normal cerebella (Ncer), 10 medulloblastoma (MD), 10 malignant glioma (Mglio), 10 Rhabdoid tumor (Rhab), and 6759 genes/reporters. The data was normalized using the first sample as a reference, i.e. dividing all samples with this reference sample, and the obtained quotients log-transformed. The AML dataset described by Bullinger et al. (2004) was downloaded from the Gene Expression Omnibus (www.ncbi.nlm.nih.gov/geo/, accession number GSE425) to contain 6283 genes/reporters. Pretreatment and filtering of the data were as in (Frigyesi et al. 2006). The final data set included 4651 genes and 108 cases. The head and neck squamous cell carcinoma data set described by Chung et al. (2004) was downloaded from the Gene Expression Omnibus (www.ncbi.nlm.nih.gov/geo/, accession number GSE686). Pretreatment and filtering of the data were as in (Frigyesi et al. 2006). The resulting data comprised 8620 genes and 53 cases. The time series data described by Chang et al. (2004) and the lung cancer data set described by Garber et al. (2001) were downloaded from the Stanford Microarray Database (http://smd.stanford.edu/index.shtml). Reporters for identical genes were merged and genes with at least 80% values were selected and corrected for missing values by KNN imputation using K=12 (in the case of lung cancer data) resulting in a dataset of 568 genes and 16 time points for the time series data and 5135 genes and 53 cases for the lung cancer data.

### Non-negative matrix factorization (NMF)

We assume that our gene expression (microarray) data is in the form of a matrix ***A*** with ***n*** rows corresponding to genes and ***m*** columns corresponding to samples and that it is the product of two nonnegative matrices ***W*** and ***H***. The ***k*** columns of ***W*** are called basis vectors. Given a matrix ***A*** and a desired number of basis vectors ***k***, also named rank, the different NMF algorithms iteratively computes ***W*** and ***H***. We have chosen to use the algorithm of Lee and Seung (1999) which minimizes a divergence functional related to the Poisson likelihood of generating ***A*** from ***W*** and ***H***. The selection of **k** is a question of dimensionality reduction. Thus the rank must be small enough to reduce noise but large enough to retain necessary information. The method of Kim and Tidor (2003) was slightly modified, such that the residual error of ***A***, ***RE*** **= |*A*** − ***WH***| is computed for different choices of rank and compared to the residual error of ***Aperm***, ***RE*** = |***Aperm*** − ***W***(***Aperm***)***H***(***Aperm***)|, where ***Aperm*** denotes the expression matrix ***A*** with the rows (genes) permuted for every column (sample). The slope in a plot of the residual error versus rank ***k*** is a measure of how much information is lost as the rank decreases. If the slope of the residual error of ***A*** is larger than that of ***Aperm*** this indicates information present in the original data set. We thus identified the smallest rank value which still preserves additional information compared to ***Aperm***.

As the NMF finds different solutions for different initial conditions, the factorizations were repeated 100 times using the previously determined rank and evaluated according to their ***RE***. The ***W***/***H***-pair with the smallest ***RE*** was selected for further analysis. It was found from the distributions of these ***RE*** that 100 iterations were more than sufficient to find a good solution (Supplementary Fig. 1).

### Association of metagenes with tumor subtypes

We used Kruskal-Wallis’ test (a non-parametric analogue to ANOVA and an extension of the Mann-Whitney test to more than two groups) to test for difference of medians for different tumour subtypes of the metagenes. Multiple testing was corrected for by using the Holm-Bonferroni method. The results are presented as box-plots with notches that display the variability of the median between tumor subtypes. The width of a notch is computed so that box plots width notches that do not overlap have different medians at the 5% significance level.

### Consensus reordering matrices

In Brunet et al. (2004) it is assumed that factoring with rank ***k*** produces ***k*** more or less disjoint classes of samples. The choice of ***k*** is assessed using connectivity matrices and the cophenetic correlation coefficient. Samples are assigned to one of ***k*** classes according to the greatest loading of matrix ***H***. Our main objection to this method of model selection is that factoring with rank ***k*** might produce other than ***k*** classes of the samples, e.g. rank 2 may produce 4 classes if the basis-vectors are not disjoint. Thus it is not the rank that is evaluated in this way but the ability of each value of the rank to classify the samples into the same number of classes. In addition, the assignment to classes also assumes disjointness as it is only done according to the greatest loading. In keeping with Brunet et al. (4) we have chosen the cophenetic correlation of the consensus matrix for the choice of number of clusters but this method is in fact not entirely correct as the cophenetic correlation varies with the number of clusters for random data as well. For each run, a connectivity matrix ***C*** is defined with entries ***cij*** = ***1*** if samples i and j belong to the same cluster and ***cij*** = ***0*** if they belong to different clusters. The average of these connectivity matrices, the consensus matrix is calculated and visualized as a tool to assess the stability of the clustering. The cophenetic correlation coefficient ***ρcc*** of the consensus matrix is calculated. We chose to modify this method by weighting the connectivity matrix of each run according to the residual error ***REt*** of that run (***(max(RE)***−***REt)/(max(RE)***−***min(RE))***).

### Biological interpretations of metagenes

To assign genes to metagenes the weights on the W matrices were ranked and a weight threshold selected to produce on average 100 genes per metagene. This approach identifies genes that contribute the most to the original features of the data and ensures that the selected genes have a strong influence on the respective metagenes. We used two methods for biological interpretations, GO term analysis using the EASE software (Douglas et al. 2003) and gene set enrichment analysis using the GSEA software (Subramanian et al. 2005). EASE identifies significant enrichment of specific gene ontology (GO) categories in a given list of genes compared to the total list of genes. Gene symbols were used in the analyses and the top ranking genes in the respective metagens were compared with total content of genes with names remaining after the initial filtering. Multiple testing was corrected for by using the step-down Bonferroni multiplicity function supplied by the EASE software. The step-down Bonferroni correction ranks the results by the statistic in ascending order. Each value is ten multiplied by (n-rank) where n is the number of results. Corrected p-values <0.05 calculated using EASE statistics were considered significant. GSEA uses a ranked gene list as an input and calculates an enrichment score to determine to what extent a predefined gene-set is overrepresented at the extremes of the entire list. Genes were ranked according to the weights in the respective basis vectors and used as an input to a gene set enrichment analysis. We used the gene sets made available within the GSEA software. The significance of the obtained enrichments scores is estimated by a permutation test and adjusted for multiple testing. We used 1000 permutations to estimate p-values and false discovery rates. The GSEA approach is independent of any arbitrarily selected threshold to assign genes to metagenes.

## Results

**Figure 1:**
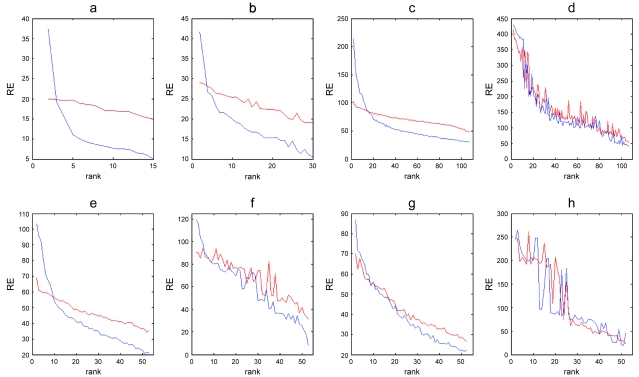
Rank estimates. **a**) The time series data, **b**) log transformed CNS data, **c**) log transformed leukemia data, **d**) raw ratio leukemia data, **e**) log transformed lung cancer data, **f**) raw ratio lung cancer data, **g**) log transformed HNSCC data, **h**) raw ratio HNSCC data. The blue line is the residual error (**RE**) for data whilst the red line is the residual error for the same data with the genes permuted (different permutations for each sample).

We estimated the rank (***k***) for each data set i.e. the number of appropriate metagenes to reconstruct the data, by evaluation of the residual error after reconstitution of the data with a given number of metagenes (Materials and Methods). To obtain non-negative matrices we used raw-ratios and log transformed ratios to which the absolute value of the lowest log value has been added. Ranks of 5–6 were found for the time series data using both log transformed (Fig. 1b) and raw ratio data (data not shown). The quality of the results for the more complex CNS, leukemia, lung, and head and neck cancer (HNSCC) data differed between the log and non-transformed data (Fig. 1c-h). Whereas the log transformed data produced smooth residual error graphs (***RE*** **=** ***|data*** − ***WH****|* as a function of ***k***) the raw ratio data showed no obvious rank. Furthermore, in contrast to the other data sets the residual error graphs for the HNSCC data was almost the same as for the randomized data indicating that NMF could not extract any informative metagenes in this data set (Fig. 1g). One possible explanation for this result could be that the HNSCC data contains more noise than the other data sets. To eliminate non-varying genes the top 20% genes with respect to variance were selected and the analysis repeated. This did however not improve the results and HNSCC was excluded from further analyses. From the residual error graphs ranks were estimated to 5, 8, 30 and 13 for the time series, CNS, leukemia, and lung cancer data, respectively, using log transformed data. The subsequent analyses were performed on log transformed data only.

### Time series data

**Figure 2:**
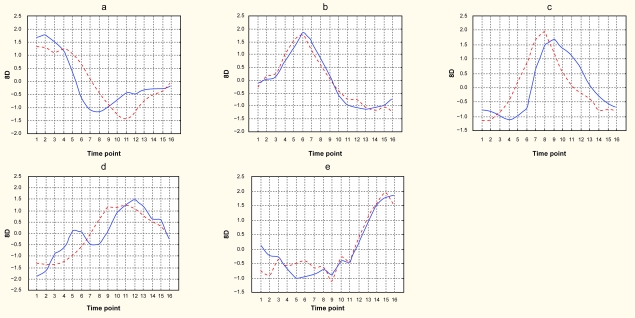
Temporal profiles for the 5 metagenes obtained from the time series data compared with temporal profiles for gene clusters obtained by a K-means 5 cluster solution. Metagenes (MG) and K-means clusters (KMC) arranged and named according to their temporal expression. To be able to compare the K-means cluster and H matrix profiles their respective values normalized across the time points and expressed in standard deviations (SD).

We calculated the ***H*** and ***W*** matrices using 100 randomized initial conditions and rank 5 and estimated the residual error for each run. The ***H/W***-pair that resulted in the smallest ***RE*** was selected for the subsequent analyses. The basis vectors were organized with respect to the order by which they are expressed during serum induction, as determined by the ***H*** weight profiles (Fig. 2). A clear temporal order of vector expression was seen. To compare the obtained results with results based global correlation K-means clustering using a predefined number of five clusters was performed. The K-means cluster expression profiles were very similar to the ***H*** weight profiles (Fig. 2) showing pair wise correlations of 0.81, 0.96, 0.67, 0.80 and 0.94 respectively. A subsequent GO analysis revealed that metagene 5 showed significant enrichment (p < 7 × 10−^9^) for GO categories related to mitotic cell cycle. The biological coherence of the K-means clusters were analyzed in a similar way and K-means cluster 1 (KMC1) was found significant (p < 0.01) for lipid biosynthesis and KMC5 for GO categories related to cell cycle and mitosis (p < 10−^9^). Taken together, the metagenes obtained by NMF showed an overall similarity to gene clusters obtained through K-means clustering.

### CNS tumors

**Figure 3:**
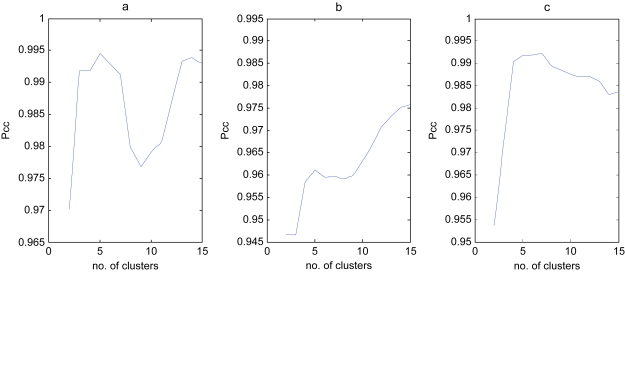
Cophenetic correlation coefficients for hierarchically clustered **a**) CNS, **b**) leukemia, and **c**) lung cancer samples. The samples were clustered using average linkage and 1-correlation as distance.

**Figure 4:**
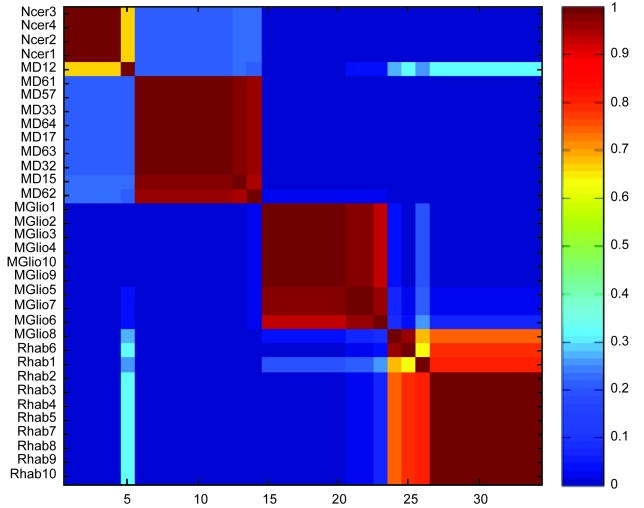
A reordered consensus matrix from a four-cluster solution of the CNS cases. The samples were clustered using average linkage and 1-correlation as distances. Deep-red color indicates perfect agreement of the solutions, whilst blue color indicates no agreement.

We calculated the ***H*** and ***W*** matrices using rank 8 and selected the ***H/W***-pair that resulted in the smallest residual error. The obtained metagenes were then subjected to GO analysis as outlined in Materials and Methods. No metagenes showed significant enrichment for GO categories when correcting for multiple testing. However, a subsequent gene set enrichment analysis (GSEA) identified significant enrichment (nominal p value <0.005 FDR < 0.1) for genes associated with favorable response to treatment (POME-ROY_MD_TREATMENT_GOOD_VS_POOR_UP) in metagene (MG) 1 and for E2A regulated genes in MG7 (GREENBAUM_E2A_UP). Metagenes 2, 4, 6 and 7 showed strong association (p < 0.05, Holm-Bonferroni corrected) with tumor subtypes or the normal samples. Post hoc analyses revealed that MG2 showed significantly (p < 0.05 using the Mann-Whitney test) higher weights in MD, MG4 in Rhab, MG6 in Ncer, and MG7 in MGlio (Supplementary Fig. 2). Hence, metagenes may show tumor subtype specificity. We then estimated the optimal number of clusters in the data i.e. molecular subtypes, using the cophenetic correlation coefficient (***ρcc***) of the consensus matrix. This analysis indicated 4–7 as an optimal number using average linkage and 1-correlation as distance (Fig. 3a). In Figure 4 a four-cluster solution is evaluated by constructing a reordered consensus matrix. The four types of samples Ncer, MD, MGlio, and Rhab are clearly separated and only two cases, MD12 and MGlio 8, are misclassified.

### Leukemia data

We calculated the ***H*** and ***W*** matrices using rank 30 (Fig. 1c) and selected the ***H/W***-pair that resulted in the smallest residual error for the subsequent analyses. Of the 30 extracted metagenes 12 showed significant enrichment for specific GO term categories (Table 1). Notably, some GO categories were enriched in more than one metagene e.g. defense response, immune response and antigen processing/presentation. We then investigated to what extent genes attached with the same GO categories was shared by different metagenes. Four metagenes showed significant enrichment of the category defense response (Table 1). Three genes were present in all four, four were present in three, 22 in two, and the majority, 74 genes (72%), was specific for individual metagenes. Three metagenes showed significant enrichment for the category immune response of which 80% of the genes were specific for individual metagenes. Hence, these metagenes represent different aspects of the same biological processes. To explore the differences in the defense/immune response profiles further all genes corresponding to these GO categories from all metagenes were used to construct a protein interaction map using the HiMAP software (http://www.himap.org/index.jsp). For exploratory reasons predicted links were used and the lowest level of significance. This produced a putative interaction network of 55 genes (Supplementary Fig. 3). When genes present in MG1 and MG4, the metagenes with the largest number of genes in the respective GO categories, were indicated in the graph it became evident that they, apart from activating overlapping regions of the network, activated genes in a sub-network specific manner.

**Figure 5:**
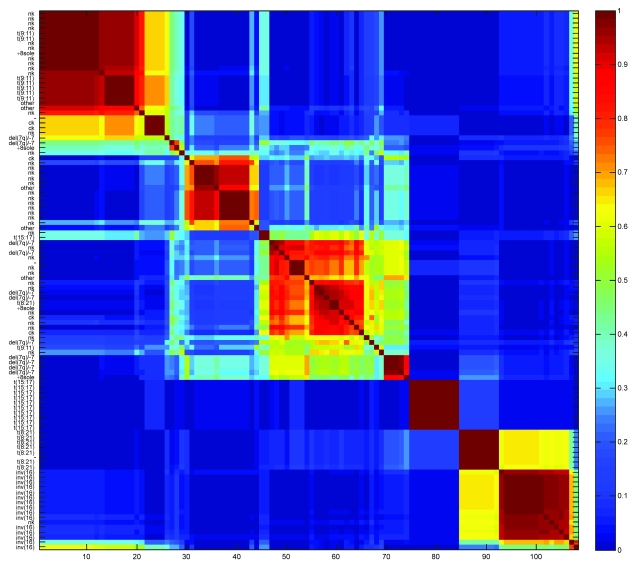
Reordered consensus matrix for a five-cluster-solutions of the leukemia data. The samples were clustered using average linkage and 1-correlation as distance. Deep-red color indicates perfect agreement of the solutions, whilst blue color indicates no agreement.

Using Kruskal-Wallis’ test and the Holm-Bonferroni method for correction of multiple tests we found that metagenes 4, 7, 9, 11, 12, 14, 15, 25 and 28 differed (p < 0.05) in their expression levels for t(15; 17), t(8; 21), del(7q)/–7, t(9; 11) and inv(16) tumors. Post hoc analyses using Mann-Whitney’s test revealed that MG7 showed significantly (p < 0.05) higher weights in inv(16) tumors, MG11 in t(8; 21), MG14 in t(15; 17), and MG25 in t(9; 11) tumors (Supplementary Fig. 4). In addition, del(7q)/–7 tumors showed significantly (p < 0.05) lower weights for MG12. To further evaluate the MG7, 14, 11 and 25 metagenes, the genes were ranked according to ***W*** matrix weights in the respective basis vectors and used as an input to a gene set enrichment analysis. MG7 showed significant enrichment (FDR < 0.001) for the ROSS_CBF_MYH gene set that distinguish the AML inv(16) subtype, MG11 significant enrichment for the ROSS_AML1_ETO gene set (FDR = 0.018) that distinguish the t(8; 21) subtype, MG14 enrichment for ROSS_PML_RAR gene set (FDR < 0.000) specific for the t(15; 17) subtype, and MG25 for the ROSS_MLL_FUSION gene set (FDR < 0.000) specific for subtypes with *MLL* (11q23) rearrangement. This is consistent with the fact that inv(16) leukemias contain *CBF/MHY*, t(8; 21) *AML1/ETO*, t(15; 17) *PML/RARA*, and that t(9; 11) leukemias contain *MLL* fusion genes. As for the CNS data, we estimated the most stable number of molecular subtypes by calculating the cophenetic correlation of the consensus matrix using increasing number of clusters (Fig. 2b). This analysis indicated 5 as an optimal number using average linkage and 1-correlation as distance. From the reordered consensus matrix shown in Figure 5 it may be seen that five fairly homogeneous clusters were formed specific for leukemias with the chromosomal aberrations inv(16), t(8; 21), t(15; 17), del(7q)/–7, and t(9; 11), respectively, in addition to two larger and more heterogeneous and unstable tumor clusters.

### Lung cancer data

**Figure 6:**
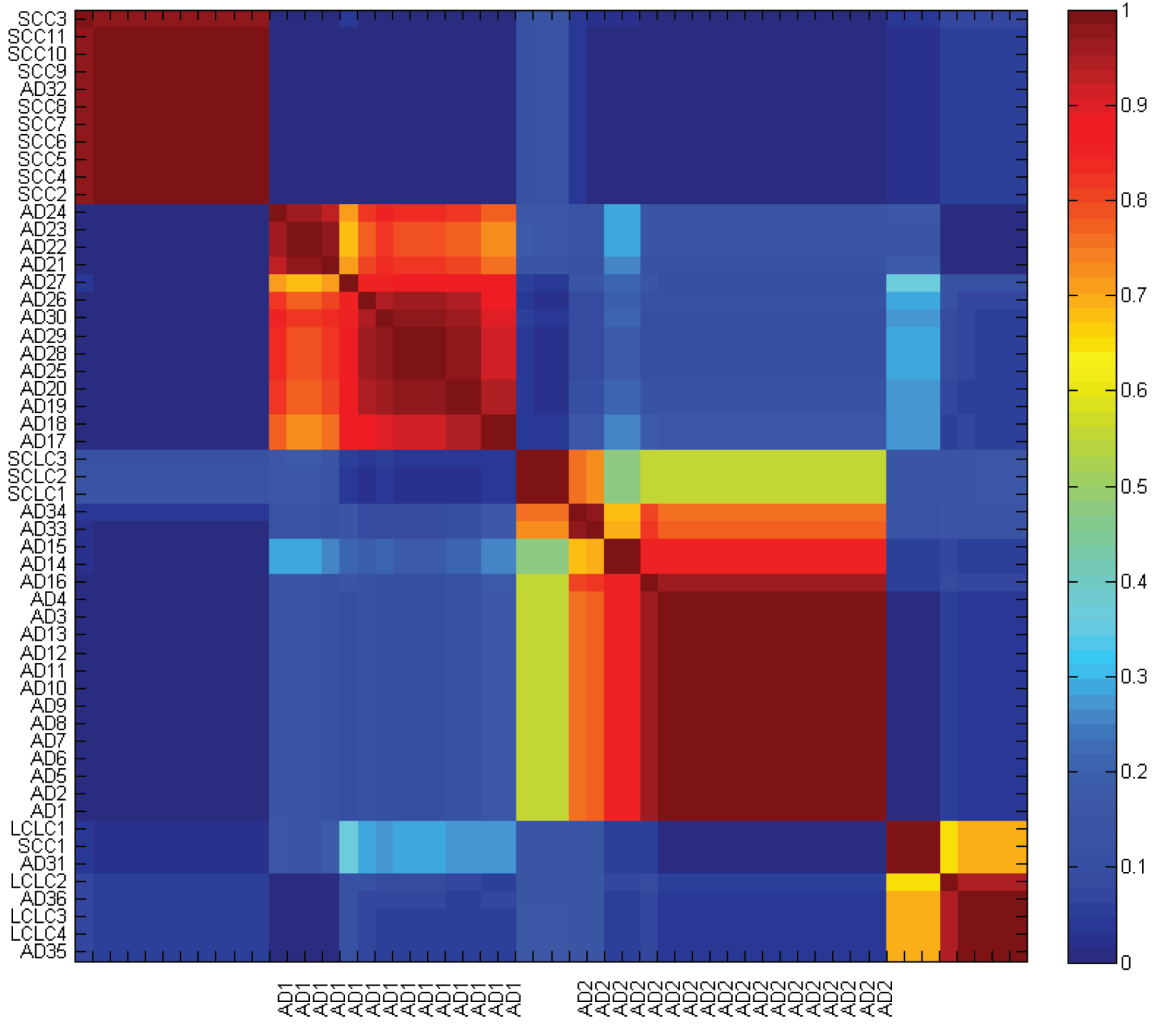
Reordered consensus matrix for a four-cluster-solutions of the lung cancer data. The samples were clustered using average linkage and 1-correlation as distances. Deep-red color indicates perfect agreement of the solutions, whilst blue color indicates no agreement.

The ***H*** and ***W*** matrices producing the smallest residual error using a rank of 13 was selected for analyses of the lung cancer data. Five of the metagenes showed significant enrichment for GO categories (Table 2). Metagenes 2 and 11 overlapped with respect to the defense response and wound healing categories and MG7 and MG13 with respect to the extracellular and extracellular matrix categories. MG2 showed 30 and MG11 50 defense response related genes with overall top ranking weights of which 20 were common to both. MG7 and MG13 shared 9 ECM related genes and contained 10 unique genes each and differed particularly with respect to collagen genes; MG7 showed top weights for the collagens 18A1, 3A1, 6A3 and MG13 for 12A1, 4A1, 5A2, and 7A1 whereas 1A2 was shared by both. These findings underscores that metagenes may influence different aspects of the same cellular processes/components. A subsequent GSEA analysis reveled that MG2 showed significant enrichment for gene sets related to cell cycle activity and bad prognosis (Table 2), MG6 showed enrichment for gene sets related to hypoxia and VEGF induced expression, and MG11 to gene sets related to epithelial-mesenchymal transition (EMT). Metagenes 2, 7, 9, 11, and 13 differed significantly (p < 0.05) in their expression levels of the four groups AD, SCC, LCLC and SCLC. Post hoc analyses using Mann-Whitney’s test revealed that MG 2 showed significantly (p < 0.05) higher weights in SCLC, MG11 in LCLC, and MG13 in SCC tumors (Supplementary Fig. 5). Hence, metagenes correspond to distinct biological processes and are associated with histological subtypes. A cophenetic correlation analysis of the consensus matrices was performed to identify stable molecular subtypes. This analysis showed best solutions for 4–7 tumor clusters (Fig. 3c). In Figure 6 a reordered consensus matrix for a four-cluster solution is presented. This analysis identified two AD subtypes, a stable cluster of SCC, and two less stable clusters composed mainly of SCLC and LCLC, respectively. The two AD subgroups differed in that MG1, MG8, and MG13 showed significantly (p < 0.05) higher weights in AD1 cases whereas MG10 and MG12 showed higher weights in AD2 cases (Supplementary Fig. 6).

## Discussion

We have evaluated the NMF algorithm as a tool to reveal biologically relevant features in complex gene expression data. NMF is a relatively new approach for gene expression analysis and no consensus procedure has as yet emerged. One issue is the use of a sparseness function as a part of the updating rules in the NMF algorithm. In essence, sparseness reduces the number of units that effectively are used to represent the data vectors and hence the number genes assigned to each metagene. Motivations for introducing sparseness as a part of the updating rules includes that all genes analyzed within in an experiment are unlikely to contribute to a specific process (Fogel et al. 2007), and that NMF without sparseness may not always yield parts based representations (Hoyer, 2004). Even if sparseness may be motivated in some situations (Gao and Curch, 2005) a side effect is that biological data of potential importance may be lost in the process. Hence, including a sparseness function in the updating rules may force the results into a biologically over-simplified model. In the present investigation we chose not to include sparseness in the updating rules and to assign genes to metagenes by an *a posteriori* selection procedure, accomplished by selecting genes with loadings above a given threshold across the whole ***W*** matrix. This approach identifies genes that contribute the most to the original features of the data and ensures that the selected genes have a strong influence on the respective metagenes.

A feature of the NMF algorithm is that only positive values are allowed in the input matrix and hence one approach would be to use raw ratios. The use of log transformation is however motivated as the relative gene expression changes, expressed as fold changes, are more likely to be of biological significance than absolute changes. In addition, raw ratios emphasize changes in the expression of high-ratio genes at the expense of changes in low-ratio genes, with a skewed influence of the factorization process as a consequence. To avoid this effect, and to equalize the scale with respect to fold changes, we transformed the log values in the input matrix to positive values by adding the absolute value of the lowest log ratio to all ratios. A possible indication that this type of data transformation may be advantageous was seen in the estimations of rank. Here we applied a modified version if the approach suggested by Kim and Tidor (2003) in which factorization solutions with increasing ranks are compared with factorizations of the equivalent randomized data. In our hands the results obtained with log transformed data were more readily interpreted than the non-transformed data.

In addition to estimate the appropriate number of dimensions, the rank estimation plots may be used to evaluate to what extent NMF captures significant structure in the data. This is an important issue as most algorithms will produce results with randomized data. The residual error graphs showed a sharp decrease in error when analyzing the original CNS, time series, leukemia, and lung cancer data compared with the slopes of the randomized data. This was in contrast to the similar slopes of the HNSCC data and the randomized HNSCC data. This is in line with the fact that CNS, leukemia, and lung cancers are defined by distinct histological subtypes whereas the HNSCC data represent a group of more homogeneous and also highly advanced tumors. However, and irrespective of the cause, the rank estimation plot for HNSCC clearly shows that NMF is not applicable to this dataset.

In Brunet et al. (2004) it is assumed that factoring with ***k*** metagenes produces ***k*** classes of samples. Our main objection to this method is that factoring with a given rank might produce more classes of samples than the number of metagenes e.g. rank 2 may produce 4 classes if the metagenes are not disjoint. In contrast to Brunet et al. (2004) we chose to perform the analysis in a two step manner by first determining the rank of the data then the optimal number of classes by cophenetic correlation of the consensus matrix. We arrived at an optimal number of 4–7 classes and 8 metagenes for the Pomeroy data. Our 4-class solution misclassified only two samples which is similar to the results obtained by Brunet et al. (2004). Analysis of the metagenes revealed that MG1 showed significant enrichment for genes associated with treatment outcome and MG7 with genes associated with the activity of the E2A transcription factor. Even though the treatment-outcome gene-set is not independent from the preset analysis, being defined by the same set of tumors, NMF extracted this gene set in an unsupervised fashion.

The analysis of the time series data gave at hand that the metagenes behaved similarly across the time points as gene clusters defined by global correlation. In this comparison we applied K-means clustering as the numbers of clusters may be pre-defined used in this algorithm, and hence a NMF solution with rank five could be directly compared with a five cluster K-means model. Both approaches assigned genes with strong enrichment for the GO category cell cycle to the last cluster/metagene. Hence, in the relatively short time series experiment analyzed, the NMF algorithm extracts essentially the same information as methods based on global correlations.

The GO analysis of the leukemia metagenes identified 12 out of 30 with significant enrichment for GO categories. Several metagenes were significant for the same GO categories indicating that metagenes may represent different aspects of the same cellular processes or components. Indeed, the tentative analysis of two of the four leukemia metagenes significant for defense/immune response showed that they represented different parts of the defense/immune response system. The analysis of the same data using global correlation detects one single cluster related to defense/immune response GO categories (Frigyesi et al. 2006). Similarly, the metagenes associated with lung cancer, MG7 and MG13, both affected the extra cellular compartment whereas MG7 influenced cell-cell contact and M13 influenced cell-cell signaling. This shows that NMF reveals biologically significant structures at a higher resolution than standard algorithms. More importantly, specific metagenes could be assigned to clinically relevant tumors subtypes. In the CNS data the four classes of samples, MD, MGlio, Rhab, and Ncer showed strong correlation with individual metagenes. In the leukemia dataset metagenes were found specific for the inv(16), t(8; 21), t(15; 17), and the t(9; 11) subtypes. This association was further validated by the GSEA analysis that showed enrichment for independent gene sets associated with *CBF/MHY*, *AML1/ETO*, *PML/RARA*, and *MLL* fusion genes in metagenes specific for leukemias with inv(16), t(8; 21), t(15; 17) and t(9; 11), respectively. Similar results were obtained for the lung cancer data in which at least three metagens could be associated with gene signatures of considerable importance for tumor behavior such as cell cycle/bad prognosis, vascularization, and epithleial-mesenchymal transition. Furthermore, SCLC, LCLC, and SCC tumors subtypes were associated with specific metagenes and the two molecular subtypes of AD could be distinguished associated with different sets of metagenes. Previously, NMF has been used to define two molecular sub-classes of SCC (Inamura et al. 2005).

Even though the NMF algorithm has been shown to perform excellently and shown to identify biologically meaningful structures at high resolution, the biological correlate of the obtained metagenes may be uncertain. It would be tempting to interpret metagenes as stable expression modules regulated by some common factors. However, as NMF produces the best solution for a given rank the composition of the basis vectors varies with rank. This is in contrast to PCA and ICA were the nature of the factors is independent of the number of dimensions (rank) chosen. However, this effect will most likely only be a challenge when working with a low number of dimensions as a change in rank will have a proportionally larger impact in these situations.

We have shown that NMF extracts biological relevant structures at high resolution in expression microarray data. Particularly, metagenes enriched for the same GO categories were shown to affect different aspects of the same biological processes. Hence, the use of NMF may contribute to a deeper understanding of tumorigenesis and tumor behavior. Several of the obtained metagenes were associated with specific tumor subtypes indicating that metagenes may constitute disease specific entities and thus that NMF may be an attractive tool for disease classification. Indeed, in a recent publication (Tamayo et al. 2007) NMF was used to extract platform independent metagens that was shown to outperform standard algorithms for classification of leukemia subtypes. Taken together NMF may be a useful approach both for interpretation of biological data and for disease classification.

## Supplementary Material

Supplementary Figure 1. Estimation of the number of iterations needed to reach a good NMF solution. For each dataset the NMF algorithm was repeated 100 times the ***RE*** recorded and then rank ordered.

Supplementary Figure 2. Association of metagenes with CNS subtypes. The p-values refer to Kruskal-Wallis’ test for difference of medians (not corrected for multiple testing). The box-plots have notches to indicate the variability of the median. Notches that do not overlap have different medians at the 5% significance level. y-axis; average H matrix loading.

Supplementary Figure 3. The protein interaction network obtained by HiMAP software (20) based on genes included in leukemia metagenes significant for the GO category immune response. Green lines indicate genes present in MG1 and blue lines indicate genes present in MG4. Insert, a sub-network common to both metagenes.

Supplementary Figure 4. Association of metagenes with leukemia subtypes. The p-values refer to Kruskal-Wallis’ test for difference of medians (not corrected for multiple testing). Only metagenes with p-values <0.01 are shown. The box-plots have notches to indicate the variability of the median. Notches that do not overlap have different medians at the 5% significance level. y-axis; average H matrix loading.

Supplementary Figure 5. Association of metagenes with lung cancer subtypes. The p-values refer to Kruskal-Wallis’ test for difference of medians (not corrected for multiple testing). The box-plots have notches to indicate the variability of the median. Notches that do not overlap have different medians at the 5% significance level. y-axis; average H matrix loading.

Supplementary Figure 6. Association of metagenes with the AD1 and AD2 molecular subtypes. The p-values refer to Kruskal-Wallis’ test for difference of medians (not corrected for multiple testing). The box-plots have notches to indicate the variability of the median. Notches that do not overlap have different medians at the 5% significance level. y-axis; average H matrix loading.

## Figures and Tables

**Table 1:** GO category analysis of leukemia metagenes.

Meta gene^1^	Go terms^2^	EASE-score^3^
1	Defense response	2.0 × 10^−12^
	Response to biotic stimulus	5.5 × 10^−12^
	Immune response	6.0 × 10^−12^
3	Defense response	7.7 × 10^−4^
	Response to external stimuli	2.1 × 10^−3^
	Response to biotic stimulus	2.3 × 10^−3^
4	Immune response	2.3 × 10^−7^
	Defense response	5.3 × 10^−7^
	Response to biotic stimulus	6.4 × 10^−7^
7	Inflammatory response	5.7 × 10^−4^
	Innate immune response	6.8 × 10^−4^
	Response to wounding	1.8 × 10^−3^
9	Antigen processing/exogenous antigen via MHC class II	1.6 × 10^−6^
	Antigen presentation/exogenous antigen	1.6 × 10^−6^
	Antigen processing	7.3 × 10^−5^
11	Defense response	3.7 × 10^−5^
	Response to biotic stimulus	1.3 × 10^−4^
	Inflammatory response	1.7 × 10^−3^
14	Copper ion homeostasis	6.6 × 10^−7^
	Heavy metal sensitivity/resistance	1.0 × 10^−5^
	Transition metal ion homeostasis	1.5 × 10^−5^
16	Hemoglobin complex	1.2 × 10^−2^
22	Nucleosome assembly	5.0 × 10^−8^
	Nucleosome	1.2 × 10^−6^
	Chromatin assembly/disassembly	1.6 × 10^−5^
24	Antigen processing/exogenous antigen via MHC class II	4.4 × 10^−3^
	Antigen presentation/exogenous antigen	4.4 × 10^−3^
	Antigen presentation	4.6 × 10^−2^
27	Antigen presentation/exogenous antigen	8.2 × 10^−12^
	Antigen processing/exogenous antigen via MHC class II	8.2 × 10^−12^
	Antigen processing	1.1 × 10^−9^
29	Mitotic cell cycle	2.1 × 10^−4^
	Nuclear division	5.2 × 10^−4^
	M phase	7.0 × 10^−4^

1 Only metagenes with significant enrichment of GO categories listed.

2 Only the top three GO categories are listed.

3 Step-down Bonferroni corrected EASE scores.

**Table 2:** GO category analysis of lung cancer metagenes.

Meta gene^1^	Go terms^1^	EASE-score^2^	Gene sets^3^	FDR^4^
2	Defense response	2.2 × 10^−9^	CANCER_UNDIFFERENTIATED_META_UP	0.000
	Response to wounding	1.3 × 10^−3^	SERUM_FIBROBLAST_CELLCYCLE	0.000
			GOLDRATH_CELLCYCLE	0.000
			CELL_CYCLE_KEGG	0.000
			BRENTANI_CELL_CYCLE	0.000
			CELL_CYCLE	0.001
			BRCA_PROGNOSIS_NEG	0.000
			VANTVEER_BREAST_OUTCOME_GOOD_VS_POOR_DN	0.003
4	Nucleus	1.3 × 10^−2^	–	
6	–	–	HIF1_TARGETS	0.001
			HYPOXIA_REG_UP	0.002
			HYPOXIA_FIBRO_UP	0.004
			HYPOXIA_REVIEW	0.021
			HYPOXIA_NORMAL_UP	0.029
			VEGF_MMMEC_ALL_UP	0.001
			VEGF_MMMEC_6HRS_UP	0.002
7	Extracellular	5.0 × 10^−12^	–	
	Extracellular matrix	8.4 × 10^−5^		
11	Defense response	2.2 × 10^−13^	TGFBETA_EARLY_UP	0.000
	Response to wounding	1.9 × 10^−3^	TGFBETA_ALL_UP	0.000
		3.5 × 10^−3^	EMT_UP	0.010
	Cell communication		CELL_MOTILITY	0.031
			JECHLINGER_EMT_UP	0.048
13	Extracellular	3.2 × 10^−10^	–	
	Extracellular matrix	3.6 × 10^−6^		

1 Only the top three GO categories are listed.

2 Step down Bonferroni corrected EASE scores.

3 Only related gene sets are listed.

4 FDR estimated using permutation tests included in the GSEA software.
